# Early post-hatch feeding and drinking behavior and body weight development in white and brown layer chickens

**DOI:** 10.1016/j.psj.2026.107896

**Published:** 2026-10-05

**Authors:** Zhanyue Wu, Henry van den Brand, Bjorge Laurenssen, Monique Ooms, Ilona van den Anker, Teresa Johansson, Ingrid C. de Jong, Roos Molenaar

**Affiliations:** a Adaptation Physiology Group, Wageningen University and Research, Wageningen, 6700 AH, The Netherlands; b Wageningen Livestock Research, Wageningen University and Research, Wageningen, 6700 AH, The Netherlands

**Keywords:** Layer chicken, Eating, Drinking, Behavior, Performance

## Abstract

Effects of early post-hatch feed and water deprivation on eating and drinking behavior and body weight development have been mostly investigated in broiler chickens. However, little is known about these aspects in layer chickens. This study investigated the eating and drinking behavior of white (Dekalb White) and brown (Lohman Brown Classic) layer chickens early post-hatch, as well as the feed and water intake and body weight development. A total of 352 in-ovo sexed female eggs on day 18 of incubation were allocated to 16 pens (8 pens per strain, 22 eggs per pen) and hatched in the pen. The first eating and drinking moment, and the eating and drinking frequency and duration in the subsequent 12 h period were determined. Feed and water consumption and body weight were measured every 6 h from hatch to 96 h of biological age. White layer chickens started eating and drinking on average at 8 and 34 h post-hatch, whereas brown layer chickens began at 5 h and 26 h, respectively. During the first 12–96 h post-hatch, each chicken consumed approximately 15 g of feed and 25 g of water on average. White layer chickens consumed more water than brown layer chickens, while feed intake did not differ. Both strains spent approximately 2 % of their time eating and drinking within the first 12 h after their first eating and drinking post-hatch. White layer chickens exhibited a higher drinking frequency and longer drinking duration than brown layer chickens, whereas hatch moment did not affect these variables. Body weight of both strains decreased during the first 30 h post-hatch and returned to its initial value at approximately 60 h post-hatch. It can be concluded that layer chickens start eating and drinking soon after hatching, but the frequency of eating and drinking remains low thereafter. Layer chickens initially lose body weight post-hatch even when feed and water are available immediately post-hatch. The exact timing of the first eating and drinking event and subsequent drinking behavior appears to be strain dependent.

## Introduction

Chickens generally begin to hatch at approximately d 19 of incubation and remain in the incubator up to 21 d of incubation (Maatjens et al., 2014). Following pulling from the hatcher, different processing and transportation procedures result in a period during which conventional chickens do not have access to feed and water (Noy and Uni, 2010). Deprivation of feed and water after hatch can have negative effects on growth and development, health, and welfare in chickens that can last until later life (Maiorka et al., 2003; Juul-Madsen et al., 2004; El-Husseiny et al., 2008; De Jong et al., 2017). Although newly hatched chickens can start eating without the presence of a mother hen, they have to develop the ability to distinguish feed from non-feed items (Hunt et al., 1967; Hogan, 1984; Edgar et al., 2016). A period of exploration, pecking, and swallowing is therefore required in the post-hatch period to learn eating and drinking (Hogan, 1984). Additionally, chickens seem to need time to recover from the hatching process. Jackson et al. (2025) investigated the behavior of broiler chickens in the first 3 d post-hatch and found that they spent more than 40 % of their time sleeping. This might imply that a certain period of rest is needed after hatching before chickens start eating and drinking. Broilers start to peck at feed and water within the first two days post-hatch, and the majority of behavior during the time that they are active is feed-related (Bizeray et al., 2000; Nielsen et al., 2010). The proportion of broilers with full crops reached 50 % at approximately 30 h of biological age when feed and water were available immediately post-hatch (Boyner et al., 2021). In addition, at 58 h post-hatch, all early feeding broilers (both fast- and slower-growing broilers) had filled crops (Smets et al., 2026). In addition, broilers that had immediate access to feed and water started to consume water earlier than feed, with 100 % of broilers drinking by 36 h post-hatch (Smets et al., 2026). Although some studies have been conducted in broilers, studies in layer chickens are largely lacking. There may be large differences as layer chickens appear to exhibit less active behavior, including eating, drinking, and foraging, than broilers during especially the first 4 d post-hatch, during which feeding and drinking behaviors are initiated (Malleau et al., 2007). This indicates there are likely differences between layer chickens and broilers, making it important to investigate the early post-hatch eating and drinking behavior in layer chickens.

Post-hatch eating and drinking behavior may be affected by several factors, including strain (Nielsen et al., 2010) and hatch moment (Boyner et al., 2021). It has been demonstrated that white and brown layer chickens differ in their behavior, and white layer chickens are in general found to be more fearful and less explorative than brown layer chickens (Rentsch et al., 2023), which may influence post-hatch eating and drinking behavior. At 20 h of biological age, late-hatched broilers showed more eating-related behavior than early hatched broilers, whereas they exhibited less eating-related behavior at 35 and 40 h of age (Boyner et al., 2021). In addition, Nielsen et al. (2010) showed that early hatched broilers had a higher eating percentage compared with mid-term and late-hatched broilers during the first 2 d post-hatch. So, it appears that the hatch moment can affect eating and drinking behavior, although studies have shown ambiguous results.

Early post-hatch eating and drinking behavior probably affects feed and water intake, body weight, and is therefore closely related to growth and welfare (Manning et al., 2007). Almost all studies related to feed and water intake in the early post-hatch period are conducted in broilers. It has been investigated that even with immediate access to feed and water after hatching, body weight does not increase immediately, and even shows an initial decreasing trend in broilers at the first 2 d post-hatch (Hogan, 1971), probably indicating that eating and drinking are limited during this period. Despite numerous studies investigating eating and drinking behavior, feed and water intake, and body weight across different breeds and developmental stages, knowledge regarding the early post-hatch stage of layer chickens remains limited. Therefore, this study aimed to investigate the first moment of eating and drinking, and the eating and drinking bouts in the subsequent 12 h post-hatch in layer chickens. Additionally, the feed and water intake and body weight in the first 96 h post-hatch for both white and brown layer chickens with feed and water available immediately were determined, taking into account their strain and biological age.

## Materials and methods

### Experimental design

The experimental protocol was approved by the Institutional Animal Use and Care Committee of Wageningen University (Approval number: NAE_2024.W-021). The experiment was carried out at the experimental facility of Wageningen University and Research, Wageningen, the Netherlands. In total, 352 in-ovo sexed female fertile eggs of a white layer chicken strain (Dekalb White, parent flock age: 36 wk) and a brown layer chicken strain (Lohman Brown Classic, parent flock age: 33 wk) were used. Eggs were divided over 16 pens (8 pens per strain, 1 m^2^ each, 22 eggs per pen) in one poultry room. The eggs were placed in the pens, and measurements were performed until the chickens reached a biological age of 96 h post-hatch.

### Incubation and housing

First-grade hatching eggs were incubated in a Petersime setter (Zulte, Belgium) under standard environmental conditions at a commercial hatchery. The start of incubation was defined as the moment when the incubation temperature reached 37.8 °C. On d 11 of incubation, all eggs were scanned using an ORBEM machine (Munich, Germany) to remove unfertilized, dead, and male embryos. At d 18 of incubation, 352 eggs were transported for 1.5 h to the experimental facility. The temperature inside the vehicle was 36 °C during transport. The average eggshell temperature (EST, °C) was 34 °C for eggs from white layer hens and 33 °C for eggs from brown layer hens.

Upon arrival at the experimental facility, eight metal-fenced pens (2 m × 1 m each) were divided in the middle with a plastic mesh into two experimental pens (1 m × 1 m each), resulting in 16 experimental pens in one poultry room. White and brown eggs were alternately placed on each side of the plastic mesh division (Fig. 1). Each pen contained wood shavings as litter, and a hatchholder (One2Born, Uden, the Netherlands) containing 22 eggs was placed in the center of the pen. After placement of the eggs, two eggs in four pens of each strain received a temperature sensor (NTC Thermistors: type DC 95; Thermometrics, Somerset, UK), which was placed at the center of the egg to measure the EST. Before the egg was placed, the room temperature was set to 34 °C, and the floor temperature was 28 °C. Afterwards, the room temperature was adjusted twice a day to maintain an average EST between 36.7 and 37.8 °C during the incubation period. The EST was recorded every 10 min until the start of hatching, after which the sensors were removed from the eggs. The relative humidity was maintained between 40 % and 60 % from egg placement until the end of the experiment.

**Fig. 1:**
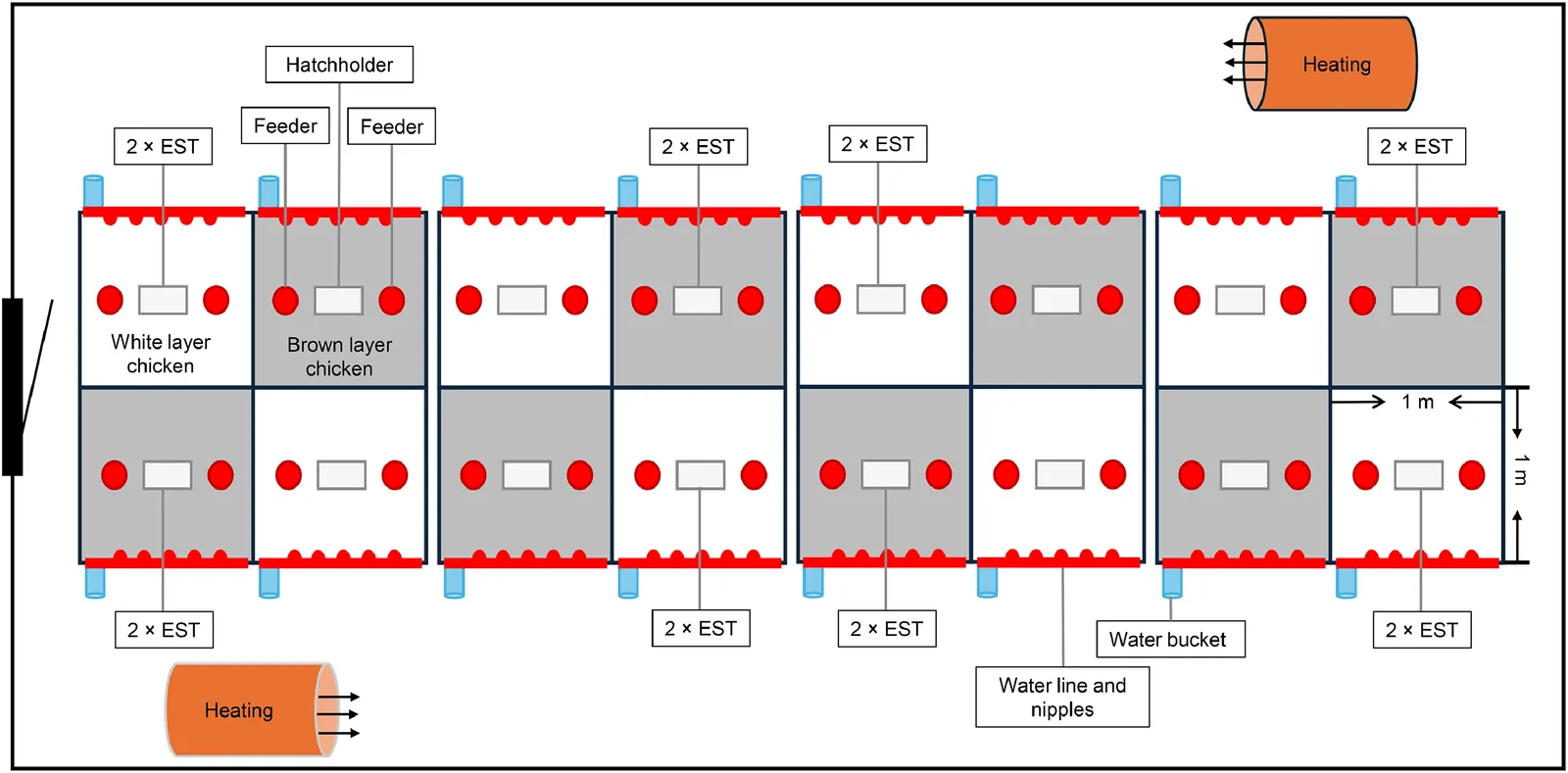
Overview of the poultry room with pens (white rectangles for white layer chicken and gray rectangles for brown layer chicken), drinking nipples and water containers, two feeders, and a hatchholder. In eight pens, two eggs per pen were equipped with an eggshell temperature sensor (2 × EST). Two heating devices were placed in the room.

Eggs were weighed at placement and continuously checked every 30 min from d 19 of incubation onward to determine whether any had hatched. Hatched chickens were weighed, tagged, and colored (head, tail, or both) for individual identification within 30 min after hatch and then returned to their original hatching location. A commercially available starter diet (pellet size 2 mm, 2,574 ME/kg, 18.0 % CP, 9.46 g/kg dLys) was provided in two open plastic red feeders (19 cm diameter) with 200 g feed each initially. Feeders were placed on opposite sides of the hatchholder. A lid with eight holes was placed on the feeder from the third day after the first chicken hatched per pen onward to prevent feed spillage. Water was available via one drinking line with five drinking nipples with a drip cup, which was connected with a water container containing 6 L of water at the onset of the experiment. Nipples were adjusted in height on a daily basis. Continuous light was provided to facilitate recording of behavior and find feed and water (range 54 to 75 lux on chicken level). Vaccinations (including in-ovo vaccinations) were not provided in this study.

### Measurements

#### Eating and drinking behavior

Each pen was continuously recorded by a camera (Hikvision, type DS-2CD204G2-1, 2.8 mm, The Netherlands) placed above the pen and the recordings were stored at 25 frames per second on a video recorder (Hikvision, type DS-7716NXI-I4/16P/S, The Netherlands). The behaviors scored were eating, drinking, pecking at feed, and pecking at water as defined in Table 1. The first eating and drinking time was scored individually relative to the moment of hatching. All hatched chickens in each pen were observed. The time range between the first and last individuals to initiate eating and drinking after hatching was also scored. Pecks on feed and water before first eating and drinking (according to the definition of eating and drinking in Table 1) were scored. Additionally, for each pen, one chicken from the earliest 10 % (early hatched), two chickens from the midpoint (mid-hatched), and one chicken from the latest 10 % (late-hatched) within the hatch window were selected to score all eating and drinking bouts over a 12-h period following the first eating and drinking time. The number of eating and drinking bouts within 12 h was expressed as eating and drinking frequency. Eating and drinking duration was expressed as the proportion of time spent eating and drinking during the 12 h measurement. Video observations were manually conducted by seven observers. The inter-observer reliability among the seven observers was assessed using 24 training videos and by calculation of the Intraclass Correlation Coefficient (ICC). The ICC results indicated a high level of agreement among observers for all scored behaviors (> 0.92).

**Table 1:** Ethogram used to determine the moment of first eating and drinking, eating and drinking frequency and duration during the 12-h period following the first eating and drinking in white and brown layer chickens.

Code	Behavior	Description of the behavior
E	Eating	Pecking at the feed in the feeder at least three times within 10 seconds. A feeding bout ended when a chicken walked away from the feeder, stopped active feeding behavior, or began to perform another activity. Movement of the chicken to another part of the same feeder was still considered part of the same feeding bout. Consecutive pecks at the feed with intervals less than 10 seconds were regarded as belonging to the same feeding bout, whereas an interruption of more than 10 seconds was considered the end of a feeding bout.
D	Drinking	Pecking at the nipple drinker or drip cup at least three times within 10 seconds. A drinking bout ended when the chicken walked away from the nipple or water cup, stopped active drinking behavior, or began to perform another activity. Consecutive pecks at the nipple with intervals less than 10 seconds were regarded as part of the same drinking bout, whereas an interruption of more than 10 seconds was considered the end of a drinking bout. When a chicken moved from one nipple drinker or drip cup to another within 10 seconds, it was still regarded as part of the same drinking bout.
PF	Pecking at feed	The chicken was considered to be pecking at feed when it pecked at the feeder without meeting the criteria for eating.
PW	Pecking at water	The chicken was considered to be pecking at water when it pecked at the nipple drinker or the drip cup without meeting the criteria for drinking. Pecking on the wood shavings in water cups was not considered pecking at water.
OP	Out of the pen	It was recorded when the first chicken of the pen was removed from the pen for group weighing.
IP	In the pen	It was recorded when the last chicken of the pen was returned to the pen after group weighing.
OA	Other	All other behaviors.

#### Feed and water consumption and body weight

The feeders and water containers per pen were weighed on a 6-h interval. Feed and water were replenished to 200 g and 6 L, respectively, when there was less than 150 g of feed or 5 L of water left during weighing. Since hatching moments differed between pens, the first measurement of feed and water after the hatching of the first chicken in each pen was defined as 0 h. The feed and water consumption data from the first 12 h after hatching of the first chicken per pen were not included in the calculation of feed and water consumption as volumes were too low to measure accurately. Two control feeders and water containers were placed in the same room outside the pens and were measured every 6 h as well to calculate weight loss due to evaporation. The evaporation was not included in the final calculation of the feed and water consumption, because the average evaporation rate from both control feeders and the water containers was below 0.01 % per 6 h. Chicken body weights were measured individually every 6 h from the first hatched chicken until all chickens reached 96 h of biological age.

#### Hatchability and hatch window

Second-grade chickens (Willemsen et al., 2008), as well as those that died and were culled during the experiment, were removed from the experiment, and their number and possible reasons were recorded. At the end of the experiment, the hatchability of each strain was calculated by dividing the hatched chickens by the number of fertile eggs placed in the pen and expressed as a percentage. The hatch window was calculated as the duration between the first and last hatched chicken in each pen.

### Statistics

All data were analyzed using SAS (Version 9.4, 2013, SAS Institute Inc., Cary, NC). Normality of residuals was assessed and variables that did not meet the assumption of normality were log- or sqrt-transformed. All transformed data presented were back-transformed and expressed as Least squares means ± SEM in tables and figures unless otherwise specified. Differences between experimental groups were considered statistically significant at *P*≤0.05. When significant differences were detected, pairwise comparisons between treatment groups were performed using the Bonferroni correction for multiple comparisons.

The first eating and drinking time was analyzed by a MIXED procedure with strain (white and brown) as a fixed effect and pen as a random effect. The eating frequency, eating duration, drinking frequency, and drinking duration in 12 h after first eating and drinking were analyzed with a MIXED procedure, in which strain (white and brown), hatch moment (early, mid, and late), and their interaction were included as the fixed effects and pen was included as a random effect. The individual chicken was considered the experimental unit in both analyses. The time range of first eating and drinking was averaged per pen and expressed as the mean for each strain, and the pen was considered the experimental unit. Additionally, the relationship between post-hatch first eating time and first drinking time was analyzed by a mixed model, with strain as a fixed effect and the first eating time and the interaction between strain and first eating time as covariates. Pen was added to the model as a random effect.

Feed and water consumption and body weight data per 6 h were analyzed with a MIXED procedure, where pen was the experimental unit. Time relative to hatching of the first chicken per pen, strain, and their interaction were included as fixed effects. Pen was added as a random effect for feed and water consumption, and pen and individual chicken nested within pen for body weight. Repeated measurements over time were conducted with pen as the subject for feed and water consumption, and with individual chicken as the subject for body weight. An autoregressive covariance structure (AR (1)) was used for feed and water consumption, whereas a compound symmetry (CS) covariance structure was used for body weight.

Hatchability was analyzed with a GLIMMIX procedure, with a binary distribution and a logit link. Strain was included as a fixed effect, and pen was treated as a random effect. Hatching time and hatch window were analyzed with a MIXED procedure, with strain included as a fixed effect and pen as a random effect. The experimental unit used was the individual chicken for hatching time and the pen for hatch window analysis.

## Results

### Timing of first eating and drinking

White layer chickens began eating on average 3.7 h later than brown layer chickens (*P*=0.03; Fig. 2). Similarly, the first drinking occurred on average 8 h later in white layer chickens compared to brown layer chickens (*P*=0.01; Fig. 2). In addition, there was no relationship (*P*=0.43; Fig. 3) between first eating time and first drinking time post-hatch.

**Fig. 2:**
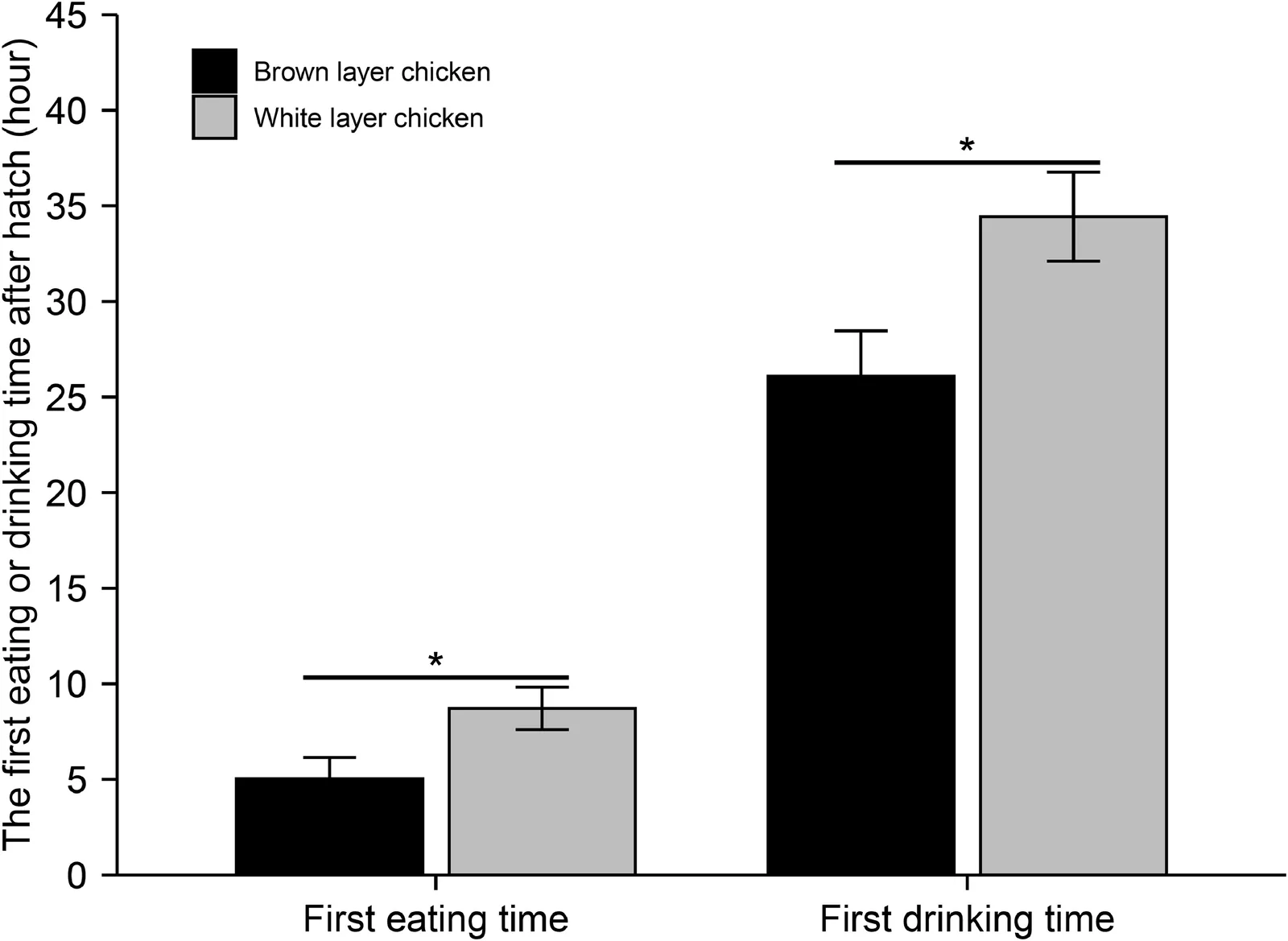
The moment of first eating and drinking of white (gray) and brown (black) layer chicken post-hatch. * Between bars denotes an effect of strain (*P*≤0.05). Pen is an experimental unit.

**Fig. 3:**
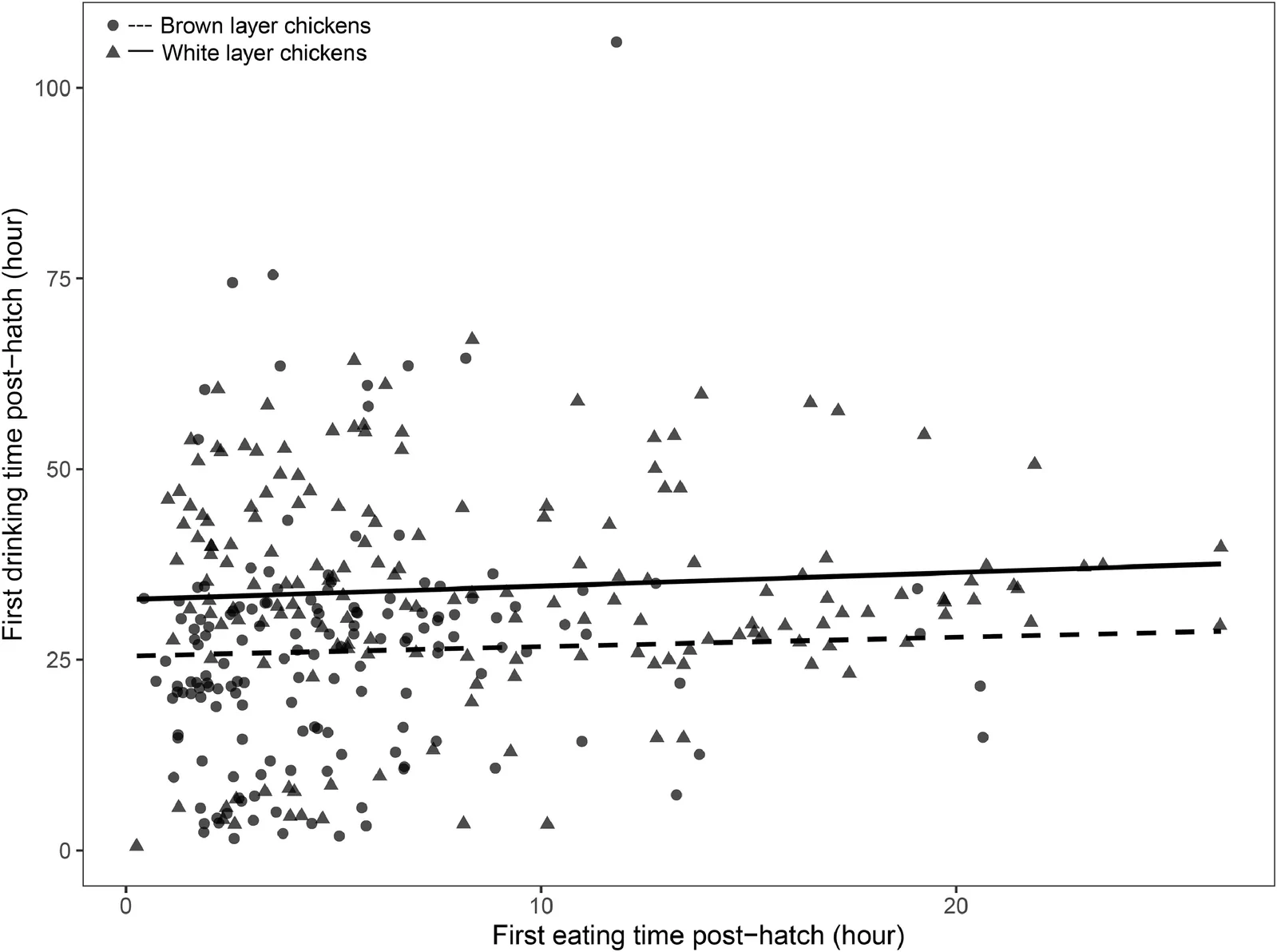
Relationship between first eating time post-hatch and first drinking time post-hatch in white layer chickens (triangle) and brown layer chickens (circle). Each point represents an individual chicken. Lines represent the predicted values from the linear mixed model, with a solid line for white layer chickens and a dashed line for brown layer chickens. Pen is an experimental unit.

In white layer chickens, the time from the first to the last individual initiating eating ranged among pens from 1.88 to 20.58 h post-hatch, whereas in brown layer chickens it ranged from 1.21 to 13.86 h (expressed as mean). Similarly, the corresponding interval for first drinking ranged among pens from 8.74 to 49.25 h in white layer chickens and from 5.51 to 54.03 h in brown layer chickens (expressed as mean).

### Eating and drinking behavior

White layer chickens showed a significantly higher drinking frequency (*P*=0.04; Table 2) and drinking duration (*P*=0.01; Table 2) in the first 12 h after first drinking compared to the brown layer chickens (Table 2). Neither eating frequency (*P*=0.24; Table 2) nor eating duration (*P*=0.49; Table 2) was affected by strain in the first 12 h after first eating.

**Table 2:** Effects of layer chicken strain (white or brown) and hatch moment (early, mid, or late) on eating and drinking frequency and duration percentage in 12 h following the first moment of eating and drinking.

Items	Eating frequency (#)	Eating duration (%)	Drinking frequency (#)	Drinking duration (%)
Strain				
White	24	0.82	40^a^	2.22^a^
Brown	10	0.23	30^b^	1.40^b^
SEM	7	0.32	8	0.37
Hatch moment^a^				
Early	14	0.38	29	1.47
Mid	17	0.53	42	2.24
Late	20	0.66	34	1.72
SEM	6	0.26	10	0.50
Strain × Hatch moment				
White × Early	20	0.62	46	2.48
White × Mid	26	0.86	39	2.46
White × Late	27	0.98	35	1.73
Brown × Early	7	0.15	12	0.46
Brown × Mid	9	0.21	45	2.03
Brown × Late	14	0.34	32	1.70
SEM	9	0.37	14	0.71
*P*-value				
Strain	0.24	0.49	0.04	0.01
Hatch moment	0.14	0.25	0.27	0.19
Strain × Hatch moment	0.81	0.85	0.13	0.10

a Behavior of four chickens per pen was observed: one early hatched chicken from the first 10 % of the hatch window, two mid-hatched chickens from the peak of the hatch window, and one late hatched chicken from the last 10 % of the hatch window. Chicken is the experimental unit (n =32 per strain).

### Feed and water consumption

Feed consumption was approximately 0.3 g per chicken in both strains between 12-30 h post-hatch and then significantly increased from 30-36 h onward (*P* < 0.001; Fig. 4), reaching 1.9 g per chicken in 90-96 h post-hatch. Neither interaction between strain and time (*P*=0.48; Fig. 4) nor strain (*P*=0.78; Fig. 4) effects were found for feed consumption. Water consumption in both layer strains showed a significant increase from 42-48 h post-hatch onward (*P* < 0.001; Fig. 4), with white layer chickens consuming 6.77 g more water per chicken over the measuring period than brown layer chickens (*P*=0.03; Fig. 4).

**Fig. 4:**
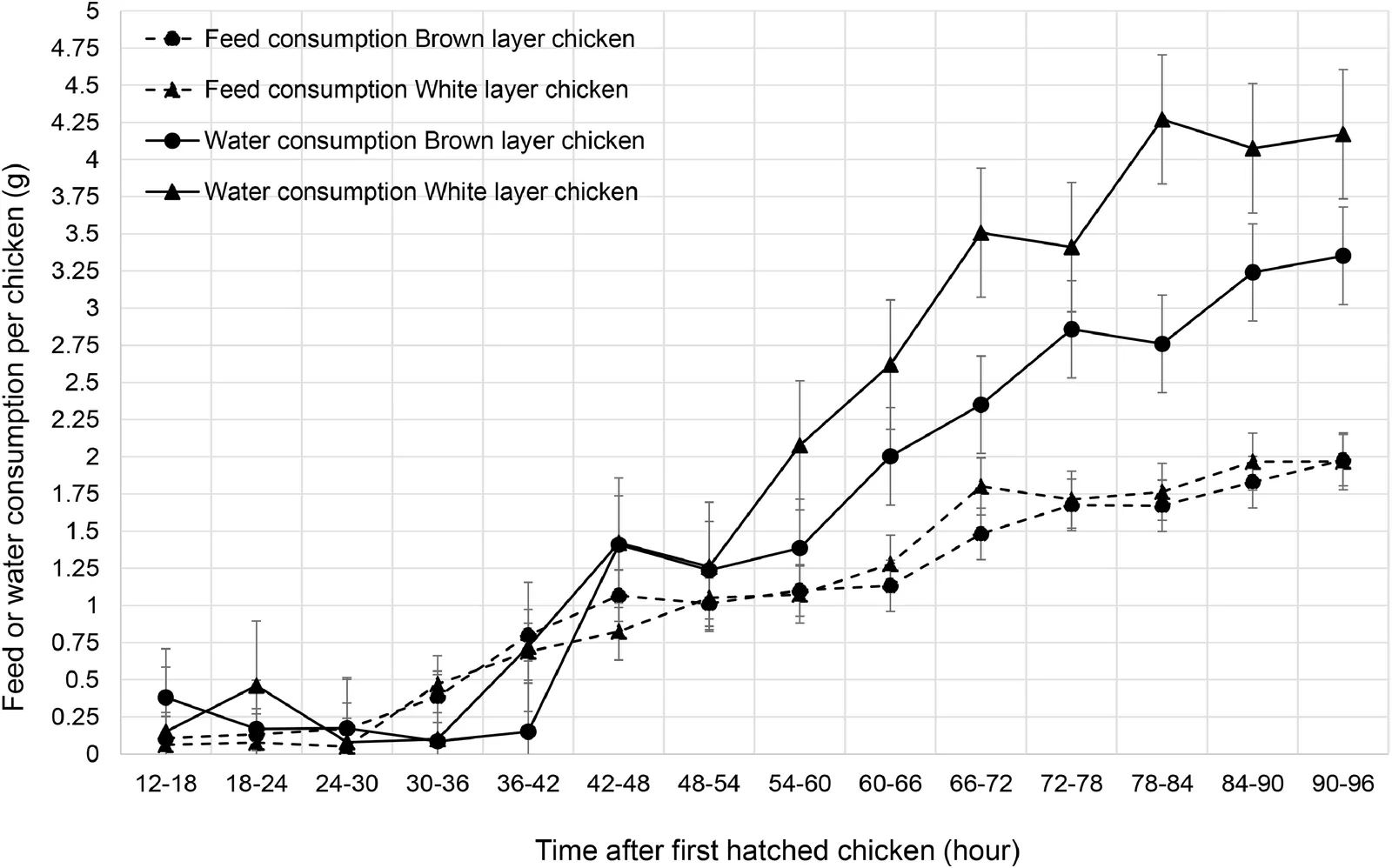
Feed (dashed line) or water (solid line) consumption from 12 to 96 h after the first layer chicken hatched in a pen. No time by strain interaction effect was found for feed or water consumption (both *P*>0.40). Feed consumption increased from 30-36 h post-hatch (*P*<0.001), but no strain difference was found (*P*=0.78). Both strain (*P*=0.03) and time (*P*<0.001) affected water consumption, with white layer chickens (triangle) drinking more than brown layer chickens (circle) and increasing from 42-48 h onward. Pen is an experimental unit.

### Body weight

An interaction between strain and time was found for body weight (*P* < 0.001; Fig. 5), with the difference between the two strains increasing over time. Body weight differed between strains at all measuring moments, except at 30 and 36 h (Fig. 5). In addition, the body weight of both strains decreased until 30 h post-hatch and increased from 42 h post-hatch onward (*P* < 0.001; Fig. 5). At approximately 60 to 66 h post-hatch, respectively, white and brown layer chickens recovered to their initial body weight.

**Fig. 5:**
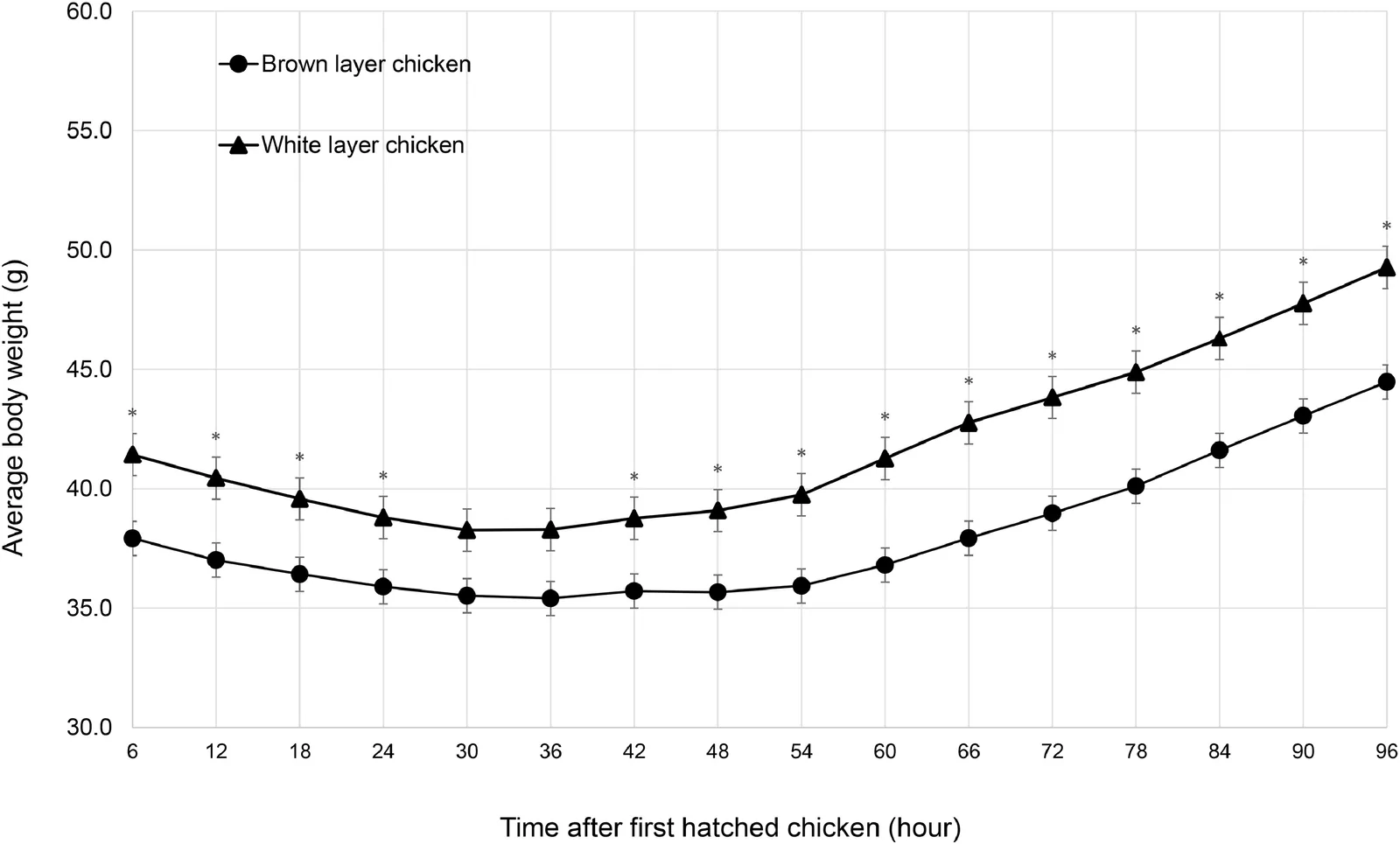
Body weight change patterns of white (triangle) and brown (circle) layer chickens after hatch. An interaction was found between strain and time (*P*<0.001). White layer chickens showed higher body weight than brown layer chickens over time, except at 30- and 36-h post-hatch. * Above each time point denotes a significant body weight difference between the two strains. Pen is an experimental unit.

### Hatch performance

The average EST of white and brown eggs ranged from 35.0 °C to 38.9 °C between placement and hatching. Mean EST was below 36.7 °C at three moments, and above 37.8 °C at one moment. The total duration that the EST was below 36.7 °C was approximately 12 h, while the period above 37.8 °C lasted approximately 9 h, accounting for 39.8 % and 26.9 %, respectively of the total time between placement and hatching moment (see Fig. 1 in the Appendix).

Egg weight at placement in the pen was 51.38 g vs 47.75 g (SEM=0.25; *P* < 0.001) for the white and brown layer chickens, respectively. The hatching moment and hatch window of the white and brown layer chickens did not differ and were on average 491.6 h (*P*=0.86; SEM=0.6) and 22 h (*P*=0.96; SEM=1.50), respectively. The hatchability of the white layer chickens was higher than that of the brown layer chickens (97.8 % vs 92.0 %; *P*=0.02). The primary cause of unhatched white layer chickens (n=4) was failure to successfully pip or hatch. The main reasons for unhatched brown layer chickens (n=14) were incomplete absorption of the residual yolk, failure to internally or externally pip, or contamination with *Aspergillus fumigatus*. During the experiment, no white layer chickens were culled. Eight brown layer chickens were culled due to low body weight gain (n=3), unabsorbed yolk sac (n=3), open navel (n=1), or leg disorders (n=1), and two chickens were found dead (data not analyzed).

## Discussion

The present experiment demonstrated that white and brown layer chickens, which had access to feed and water immediately post-hatch, performed different eating and drinking behaviors from hatch to 96 h of biological age. Both white and brown layer chickens started eating first and then drinking. The first eating and drinking time, subsequent drinking frequency and duration, water consumption, as well as body weight were strain dependent, while the eating frequency, eating duration, and feed intake did not differ between strains. The body weight of both strains decreased initially and then increased, with a gradually larger difference between the two strains.

### Timing of first eating and drinking

In this study, the moment of first eating was observed within approximately 8 h after hatching in both strains. This appears to be much earlier than found in other studies. Boyner et al. (2021) found in broilers that 5 % of the chickens were eating or were close to the feed at 25.4 h of their biological age. In the current study, the first drinking time was observed at 34 and 26 h post-hatch in white and brown layer chickens, respectively. The findings from the white layer chickens agree with Broom (1968), who found that drinking in individually reared domestic fowl chicken started at approximately 36 h post-hatch. In addition, in the current study, at approximately 20 h after hatching, all chickens had completed their first eating, whereas the onset of first drinking was delayed until approximately 55 h post-hatch. Little is known about the potential factors that may affect the start of eating and drinking in chickens after hatching, but differences in experimental design, chicken strain, rearing management, observation approach, the way in which feed and water are provided, as well as the definition of eating and drinking may affect the outcomes. For example, Boyner et al. (2021) used scan sampling, and chickens standing near the feeder were also included as showing feeding-related behavior in their study, which differs from the analysis in the current study. Scan sampling provides a representation of the overall distribution of behaviors (Hanna and Lauderdale, 2022), whereas the current study employed continuous observations to accurately identify the timing of the first feeding and drinking events for each individual. These methodological differences may have contributed to the difference between studies. Another example is the difference in preplacement holding time, which significantly affects the eating and drinking behavior (Özlü et al., 2022). In the current study, chickens had immediate access to feed and water after hatching without a pre-placement holding time. Another factor that might have affected the timing of first eating and drinking is light intensity. In the current study, the light intensity near the drinking nipple line was lower than in other areas in the pens, because the water line was installed at one side of the pen. Chickens hatch with a predisposition to peck shiny objects, which in natural environments are usually water droplets to help them find the water source (Duncan, 1998). Both white and brown layer pullets have been found to be more active at higher light intensities (Van der Eijk et al., 2026). The lower light intensity at the drinking zone may have extended the duration of exploration and search for water sources. The potential influence of all these factors highlights the need to consider them in future experiments.

In the current experiment, before the occurrence of the first eating and first drinking behavior as we defined, both white and brown layer chickens performed, on average, one peck at feed and one peck at water. These pecks did not meet the definitions of eating and drinking applied in this study, but it was also difficult to recognize in our videos whether it was an exploratory pecking or actual eating or drinking. This pecking gives insight into the process by which chickens explore and possibly recognize feed and water quite rapidly post-hatch. Although Nielsen et al. (2010) reported a prandial drinking pattern in newly hatched broiler chickens, suggesting a temporal association between feeding and drinking. No relationship between the onset of first feeding and first drinking was observed in both strains in the current study. This difference may be related to the potential breed differences between layer chickens and broilers. Additionally, chickens in Nielsen et al. (2010) were observed during the first week of age, when feeding and drinking behaviors had already become relatively mature, whereas the current study focused only on the first timing of the eating and drinking events.

### Eating and drinking behavior

In the current study, chickens spend approximately 1-2 % of their time eating and drinking throughout the 12 h after the first eating and drinking moment. Resting following hatching is critical for neonatal chickens (Malleau et al., 2007). During the first few days post-hatch, chickens spend a substantial proportion of their time sleeping, while allocating relatively little time to other activities, such as feeding, drinking, and preening (Malleau et al., 2007; Jackson et al., 2025). In addition, the yolk sac has not yet been fully absorbed and continues to supply nutrients to the chicken in the early days post-hatch (Ravindran and Abdollahi, 2021). The requirement for rest during the early post-hatch period, together with the nutritional support provided by residual yolk reserves, may contribute to the relatively low feeding frequency observed during the initial exposure to exogenous feed, although neither resting behavior nor residual yolk sac weight was measured in the current study.

Hatch moment has been demonstrated to affect eating and drinking behavior, with early hatched broilers eating more than late hatchers in the first two days post-hatch (Nielsen et al., 2010). Early hatched broilers performed less eating-related behavior at 20 h of biological age than late-hatched broilers, while late-hatched broilers showed less eating-related behavior at 30 and 40 h of biological age (Boyner et al., 2021). It can be hypothesized that late hatchers may express more eating and drinking behavior than early hatchers, because they can learn from their conspecifics (Turner, 1964; Edgar et al., 2016). However, the current study indicated that hatching moment did not affect post-hatch eating and drinking behavior. This may be partly explained by the difference in the definition of eating behavior between studies. For example, Nielsen et al. (2010) and Boyner et al. (2021) considered chickens standing on the feeder or close to the feeder as eating-related behavior, whereas in the current study, eating behavior was only recorded when the chicken pecked the feed at least 3 times within 10 sec.

### Feed and water consumption

In the current study, chickens consumed on average 14.57 g of feed and 24.89 g of water during the 12 to 96 h post-hatch period. Noy and Pinchasov (1993) showed that broilers with immediate access to feed and water consumed 1.5 g of feed during the first 24 h post-hatch, which is higher than that in the current study (0.22 g between 12 and 24 h post-hatch), which might be because broilers were selected for higher feed intake and faster growth rate (Tavárez and Solis de los Santos, 2016). Stahl and Sunde (1983) showed that layer chickens consumed on average approximately 36 mL of water per chicken in the first 12 to 96 h post-hatch when water was immediately available, which was higher than found in the current study (approximately 25 g per chicken between 12 and 96 h post-hatch). This may be due to the variations in the age and physiological state of the chickens at the time of measurement. Chickens of Stahl and Sunde (1983) were placed in the pens approximately 5 to 8 h after removal from the incubator. This implies that first hatching chickens were probably already 36 h of biological age at pulling. Moreover, they may have experienced dehydration in the incubators, which could in turn have increased the water intake when water became available.

### Body weight

White and brown layer chickens lost 8.25 % and 6.78 %, respectively, of their initial body weight in the first 30 h post-hatch and required approximately 60 and 66 h, respectively, to regain their initial body weight at 6 h post-hatch. This is in accordance with a study in red junglefowl chickens showing an initial decrease in body weight during the first two days after hatching, even when feed was available immediately after hatching (Hogan, 1971). In addition, Nitsan et al. (1991) found that the body weight of broilers increased at the first day post-hatch, which differs considerably from our results. This difference can probably be explained by the fact that Nitsan et al. (1991) used day-old chickens (probably post-pulling), whereas in the current study measurements were performed post-hatching. Additionally, Nitsan et al. (1991) used broilers, which have a higher growth rate and display more eating and drinking behavior than layer chickens (Buzala and Janicki, 2016). Overall, the evaporative water loss, the low feed and water intake, as well as the absorption of the yolk sac at the early stage, probably contributed to body weight loss in the current study. Newly hatched chickens have a wet body surface (Romanini et al., 2015), and the body weight loss in the early days is primarily due to the water loss (Hamdy et al., 1991). It has been determined that chickens lost 0.4 g body weight every 3 h post-hatch without water intake between hatching and pulling time (Van der Pol et al., 2015). Although chickens began their first eating within 8 h post-hatch and drinking later, their subsequent eating and drinking behavior and feed and water intake remained limited in the current study. The limited ability to digest and absorb feed immediately after hatching possibly further contributed to the post-hatch decrease in body weight (Nitsan et al., 1991). Pinchasov and Noy (1993) showed that body weight loss observed in chickens during the first day post-hatch regardless of the availability of feed and water is mainly attributable to the decrease of the yolk sac. The yolk sac provides nutrients to newly hatched chickens, and in some studies the absorption of the yolk sac accelerated with the exogenous feed consumption (Van der Wagt et al., 2020). The body weight of both strains increased significantly after 48 h post-hatch, accompanied by a marked increase in feed and water consumption between 30-36 h and 42-48 h, respectively, which may indicate the dominant effects of feed and water intake in body weight gain.

### Strain effect

#### First eating and drinking time

White layer chickens started approximately 3 and 8 h later, respectively, with their first eating and drinking than brown layer chickens. This strain effect may be explained by the strain difference in behavior and parent flock age. First, white layer chickens are in general more fearful than brown layer chickens, which has been demonstrated in several behavioral tests at adult age (Mahboub et al., 2004; Uitdehaag et al., 2008; De Haas et al., 2013). White layer chickens, therefore, appear to exhibit less exploratory motivation than brown layer chickens (Rozempolska-Rucinska et al., 2017), thereby perhaps also requiring more time to approach feed and water. Second, the parent flock of the white layer strain was 3 weeks older than the brown layer strain and consequently, a higher egg weight and hatch weight were observed in the white layer strain than in the brown layer strain. Chickens from older flocks often have larger yolk sacs with higher protein and fat content, as well as greater energy reserves in the yolk-free body, compared with those from younger flocks (Nangsuay et al., 2013). Therefore, it might be possible that white layer chickens had a relatively higher energy availability derived from yolk sac lipid oxidative metabolism (Hicks et al., 2019), which might have affected their initial eating activity in the current study. However, Van Marwijk (unpublished results, cited in (Van den Brand et al., 2023)) did not find a difference in the start of eating between broiler chickens originating from a 36 and 59-week-old breeder flock. In the current study, the effect of flock age, residual yolk size, and residual yolk utilization on the start of eating and drinking is not investigated.

#### Eating and drinking behavior

The drinking frequency and drinking duration in white layer chickens were approximately 33 % and 59 %, respectively, higher than in brown layer chickens in the current study. No significant differences in eating frequency or eating duration were found between strains in the current study. In terms of drinking frequency, white layer chickens started drinking approximately 8 h later than brown layer chickens, meaning that white layer chickens experienced a longer duration of water deprivation and were likely thirstier, leading to higher drinking motivation than brown layer chickens. Both broilers and layer chickens deprived of water for longer periods exhibited significantly higher water intake and spent more time drinking upon re-access to water (Sprenger and Tuyttens, 2009; Rault et al., 2016). In the first 3 d post-hatch, chickens gradually establish a link between hunger, nutritional status, and metabolic demands, which in turn drives feeding behavior (Hogan, 1984). However, in the current study, eating behavior was observed within 24 h post-hatch, a period during which feed intake remains limited and the yolk sac continues to supply nutrients (Nangsuay et al., 2013). This may explain why there were no significant differences in eating behavior between white and brown layer chickens, but our understanding of the relationship between their eating behavior and post-hatch nutritional status in the first few days post-hatch is still limited.

#### Feed and water consumption and body weight

In the first 36 h post-hatch, white layer chickens consumed slightly less water than brown layer chickens, whereas the white layer chickens drank more after that. It is widely accepted that higher feed and water intake have been associated with higher body weight gain (Aggrey et al., 2023). As discussed above, the more frequent drinking behavior and higher water consumption, together with higher egg weight, may have contributed to higher body weight in white layer chickens thereafter. In the first 36 h, average body weight loss in white layer chickens was approximately 0.6 g greater than in brown layer chickens, and white layer chickens required about 6 h less to recover their initial body weight than brown layer chickens. White layer chickens started eating later, while subsequent feeding behavior was similar in both strains. This may explain the greater body weight loss observed in white layer chickens, whereas their higher subsequent water intake may have contributed to faster body weight recovery.

### Hatch performance

The hatchability of white layer eggs was approximately 6 % higher than that of brown eggs. Appropriate eggshell temperature is critical for hatchability (Ipek et al., 2014). The eggshell temperature from placement (d 18) to hatching fluctuated for both white and brown eggs and was more than 50 % of the time outside the proposed optimal range but showed similar patterns for both strains. Therefore, the observed temperature fluctuations are unlikely to explain the large difference in hatchability between the two strains but may have affected the chicken quality at hatch (Maatjens et al., 2016). In addition, the lower hatchability and higher cull rate in brown layer chickens might be related to the presence of *Aspergillus fumigatus*, which can cause embryonic mortality, thereby reducing hatchability (Williams and Brake, 2000; Rodríguez-Ortega et al., 2021). Newly hatched chickens infected with *Aspergillus fumigatus* can develop respiratory diseases, leading to mortality in 1 to 2 d post-hatch (Jacobsen et al., 2010). Although only some brown eggs were infected with *Aspergillus fumigatus* in the current study, and no signs of infection were observed in the hatched brown layer chickens up to 4 days of age, approximately 90 % of culled brown layer chickens were found hatched in the pens where eggs affected by *Aspergillus* were found. Therefore, the contribution of *Aspergillus* contamination to the observed hatchability difference between white and brown layer chickens cannot be ruled out, but its role in post-hatch development and early eating and drinking behavior remains uncertain.

## Conclusion

In conclusion, both white and brown layer chickens start eating within approximately 8 h and are drinking at a maximum of 36 h, when provided feed and water immediately after hatch. Although chickens ate soon post-hatch, the body weight of layer chickens initially decreased for approximately 30 h before gradually increasing. In addition, the time that starts eating and drinking post-hatch, and the subsequent eating and drinking behavior, seem strain-dependent.

## Author contributions

**Zhanyue Wu** analyzed the data, conducted the literature review, prepared the data visualizations, and drafted the manuscript. **Henry van den Brand** contributed to the conception and design of the study, developed the methodology, supervised the research, and critically reviewed the manuscript. **Bjorge Laurenssen** conducted the experiments and critically reviewed the manuscript. **Monique Ooms** conducted the experiments, curated and analyzed the data, and critically reviewed the manuscript. **Ilona van den Anker** conducted the experiments, analyzed the data, and critically reviewed the manuscript. **Teresa Johansson** conducted the experiments and critically reviewed the manuscript. **Ingrid C. de Jong** contributed to the study design, secured the funding, conducted the literature review, and critically reviewed the manuscript. **Roos Molenaar** contributed to the study conception and design, conducted the experiments and the literature review, analyzed and interpreted the data, prepared the visualizations, and critically reviewed the manuscript. **All authors** explicitly approved the final version of the manuscript for publication and agree to be personally and publicly accountable for all aspects of the work, ensuring that any questions concerning its accuracy or integrity are appropriately investigated and resolved.

## Disclosures

The authors declare that they have no competing financial interests or personal relationships that could have appeared to influence the work reported in this paper.

## Appendix Supplementary materials

Appendix Figure 1. Average eggshell temperature (°C) of eggs (n = 8 per strain) from white and brown layer strains between placement in the pen and hatching.
